# Tanzanian adolescents’ attitudes toward abortion: innovating video vignettes in survey research on health topics

**DOI:** 10.1186/s12978-024-01809-x

**Published:** 2024-05-21

**Authors:** Anna Bolgrien, Deborah Levison

**Affiliations:** 1 Institute for Social Research and Data Innovation, University of Minnesota Twin Cities, 225 – 19th Avenue South, Minneapolis, MN 55455 USA; 2 Hubert H. Humphrey School of Public Affairs, University of Minnesota Twin Cities, 301 – 19th Avenue South, Minneapolis, MN 55455 USA

**Keywords:** Abortion, Adolescent, Children, Sexual and reproductive health, Survey methods, Tanzania, Vignettes

## Abstract

**Background:**

The purpose of this study was to pilot an innovative cartoon video vignette survey methodology to learn about young people’s perspectives on abortion and sexual relationships in Tanzania. The Animating Children’s Views methodology used videos shown on tablets to engage young people in conversations. Such conversations are complicated because abortion is highly stigmatized, inaccessible, and illegal in Tanzania.

**Methods:**

The cartoon video vignette methodology was conducted as a part of a quantitative survey using tablet computers. Hypothetical situations and euphemistic expressions were tested in order to engage adolescents on sensitive topics in low-risk ways. Qualitative interviews and focus groups validated and further explored the perspectives of the young respondents.

**Results:**

Results indicate that 12–17 year-olds usually understand euphemistic expressions for abortion and are aware of social stigma and contradictory norms surrounding abortion from as young as age twelve. Despite the risks involved with abortion, this study finds adolescents sometimes view abortion as a reasonable solution to allow a girl to remain in school. Additional findings show that as adolescents wrestle with how to respond to a schoolgirl’s pregnancy, they are considering both the (un)affordability of healthcare services and also expectations for gender roles.

**Conclusions:**

Digital data collection, such as the Animating Children’s Views cartoon video vignettes used in this study, allows researchers to better understand girls’ and boys’ own perspectives on their experiences and reproductive health.

## Abstract (plain language summary)

The Animating Children’s Views project used cartoon video vignettes to collect quantitative and qualitative data on girls’ and boys’ (infrequently included) perspectives about this sensitive topic as these young people aged into and figured out how to navigate sexual maturity in rural and urban Tanzania. This novel survey technique leveraged digital technology to better engage young people’s perspectives about sensitive health topics. Despite the risks involved with abortion, this study finds adolescents sometimes view abortion as a reasonable solution to allow a girl to remain in school. Additional findings show that as adolescents wrestle with how to respond to a schoolgirl’s pregnancy, they are considering both the (un)affordability of healthcare services and also expectations for gender roles. We argue that digital data collection allows survey research to include girls and boys, to better understand how reproductive health outcomes are inextricably linked to their future lives.

## Introduction

*“Sometimes bad luck happens, and a girl gets pregnant when she is still studying. And if the teacher knows about it, she might not attend school. And she can either abort the pregnancy or deliver and care for the baby. Some of the students are curious. They might know she is pregnant. It will be a shame on her. They can report her to the teacher, and she might be dismissed from school. The teachers will not understand her situation; they will not know it’s something that you did not plan for. They will feel you did it deliberately.”* [girl, age 15, focus group, urban]

Approximately one-quarter of Tanzanian adolescents girls become pregnant between the ages of 15 to 19 years [43]. Many young people in Tanzania are sexually active from as young as 10–14 years of age or plan to become sexually active before marriage [27, 15]. Some girls have sex because boys are not expected to be abstinent, and they expect their girlfriends to have sex with them. In addition to seeking sexual relationships out of desire, girls may enter into consensual transactional sexual relationships with older boys or men as a way to secure financial stability [25, 46]. Peer pressure, lack of familial financial support, lack of information about health services, and poverty all strongly correlate with high rates of teenage pregnancy [ 29]. Cultural barriers make it difficult for schools, non-governmental organizations, and parents to communicate appropriate and accurate reproductive health information to youth [34, 45]. Girls may face difficulties obtaining and paying for contraceptives [23] or for an abortion after an unplanned pregnancy.

Girls experience stigma at multiple levels of society when navigating teenage sexual relationships and their education [18, 33]. To surmount these and other challenges, Tanzanian girls and boys are convinced that “education is the key to life” (*Elimu ni ufunguo wa maisha.* See Vavrus [44]). Becoming pregnant while in school puts a girl at risk of social isolation and being labeled a “bad girl” [11]. Additionally, at the time of this study the legal reality was that pregnant girls were expelled from school [8]. In 2021, the World Bank’s influence and change in Tanzanian leadership have led to changes in the government’s approach to schoolgirl pregnancy and motherhood (Reuters [35]). However, implementation of the revised policy requires separate schools for young mothers, which are unlikely to be accessible for much of the population any time soon.

What results is a culture of secrecy where young people hide their relationships from parents and peers alike. Abortion – illegal in Tanzania – may seem like a way of escaping a life-long penalty for premarital sexual activity. Yet, unsafe abortions account for a substantial fraction of maternal deaths in Tanzania [17]. Anti-abortion sentiment arises from religious objections; pro-choice discourse from public health aims to reduce maternal mortality rates due to unsafe illegal abortions; and human rights organizations call for women to have a choice in their reproductive health [36]. In the event of a pregnancy, secrecy can be maintained only through unsafe and potentially deadly abortion services, as the great majority of girls cannot raise the necessary funds for safer illegal abortions in private clinics [39].

Given the consequences of the lack of social support, accurate health information, and significant impacts to their lives, it is important to learn more about how young people are navigating the competing pressures of engaging in sexual relationships and staying in school if policy makers and researchers are to help improve outcomes for adolescents. While the overall study was methodologically driven and featured various topics and themes relating to the lives of adolescents in Tanzania, this paper focuses on how a cartoon video vignette methodology engaged young people in order to learn about how they weighed the risks and benefits of abortion in a context where teen pregnancy may be the end of education for girls.

In this paper, we present mixed-methods results using quantitative and qualitative data collected in response to a story about teen pregnancy. We find children and adolescents understood concerns about social stigma and were aware of contradictory norms surrounding abortion. They also shared their perceptions of inadequate health care services and views on gendered decision-making. Our results show that adolescents were considering complex social, medical, ethical, and pragmatic factors surrounding teenage pregnancy and abortion. In addition to the substantive findings, this paper also argues that a vignette methodology can be a useful way to collect survey data on children’s perspectives, made possible through advances in the usability and affordability of digital technology in field work. We describe two techniques – the use of euphemistic expressions and asking questions about hypotheticals – to learn children’s opinions about sensitive topics like abortion.

### Methodology: using video vignettes in survey research

The relationship between high rates of teenage pregnancy and unknown rates of abortion – both sensitive topics – is examined primarily in qualitative research because researchers can take more time to establish rapport and build trust, thereby reducing risks to and vulnerability felt by participants [9]. Qualitative research is ideal for understanding nuances of how youth are interpreting cultural norms towards abortion and how they are thinking about access, effectiveness, and safety of abortion services in the event that they may at some point face an unplanned pregnancy. However, qualitative studies of youth’s experience and attitudes towards abortion across sub-Saharan Africa typically engage with older girls, most often between 15 and 24 years old, such as Bajoga et al. [3] and Otoide et al. [31] in Nigeria, Silburschmidt and Rasch [39] in Tanzania, Hall et al. [11] in Ghana, and Marlow et al. [22] in Kenya. Data on boys of all ages and younger girls are limited; one exception is Sommer et al. [40].

It can be difficult to gather quantitative data on experiences of abortion. Survey research on abortion in Tanzania and elsewhere generally focuses on adult women and occasionally men. While married or older women may feel less stigma associated with sexual behaviors, survey respondents may still feel uncomfortable responding to abortion-related topics that may be inappropriate to discuss in public, topics that would lead to admitting an illegal action, or topics where a truthful answer would be a violation of a social norm [42]. In the case of abortion, qualitative interviews with adult women in Tanzania and elsewhere who have experienced abortion frequently report that internalized stigma results in abortions being underreported or omitted from survey data (e.g., Astbury-Ward et al. [1] for the UK; Haws et al. [13] for Tanzania). Quantitative surveys typically avoid sensitive topics, particularly in contexts where privacy may be impossible. This is of high importance when engaging with vulnerable people. Adolescents may be particularly alert to sensitive topics and not feel comfortable disclosing their experiences in direct conversation [4]. If the children or adolescents are overheard saying anything that an adult deems inappropriate, they could be physically punished, have food withheld or be otherwise penalized. Quantitative studies of adolescents in Tanzania include a few examples of young people’s sexual experiences but these do not specifically discuss abortion [32, 28, 38].

Vignettes are one way that survey researchers can learn respondents’ views, by asking them about characters in a story instead of about personal experiences [10, 30, 14]. Vignettes can be written text, cartoons, read-aloud, or videos; respondents answer questions based on details in the story. Videos shown on tablets are similar to methods of communication that many young people in Tanzania are familiar with: 100% of our respondents had seen a video before. Instead of solely using a traditional question-answer format – which may feel to adolescents like an examination – videos creatively allowed participants to engage with stories.

Vignettes about abortion have been used in previous studies with adults (Sastre et al. [37] in France, Hans and Kimberly [12] in USA, Kavanaugh et al. [16] in Nigeria and Zambia). In a study in neighboring Kenya, Mitchell et al. [24] used vignettes to compare adolescents’ recommendations to a fictional couple, their own hypothetical future, and real examples of peers’ unplanned pregnancies and abortions. Their results suggest that respondents held different expectations for the vignette couple than for themselves or their peers.

The research presented in this paper fills a gap in the literature: young people’s perspectives – especially those of younger girls and of boys – are often excluded from research on abortion and sexual relationships. We show results from a novel methodology designed to illuminate the perspectives of children in low-income countries while reducing participation risks for young respondents.

### Methodology: Animating Children’s Views (ACV)

The Animating Children’s Views (ACV) methodology developed by Levison and Bolgrien [21] used cartoon vignettes to present short stories to 12-to-17-year-olds in rural and urban northern Tanzania. While the use of tablet computers to collect survey data in the field is not a new technology, the ability to incorporate short videos during the survey allowed the field team to better engage young respondents during the interview. Respondents watched the cartoons and then responded to survey questions posed by interviewers about the situation and possible outcomes for characters in the stories. The innovation in using tablets to show videos establishes a way to create an experience where the respondent is expressing perspectives or opinions on a qualitative topic, but responses are coded as quantitative data collected during a survey.

**Fig. 1:**
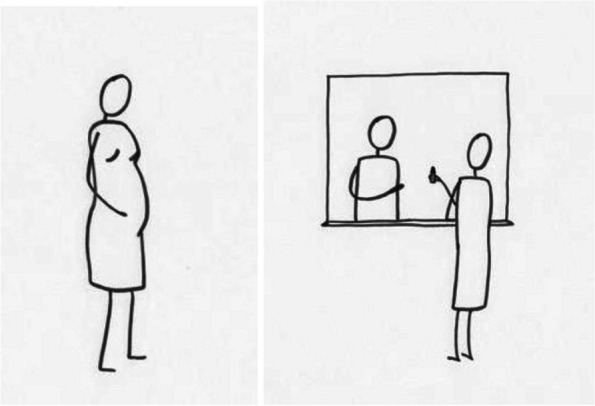
Images of pregnant girl and “getting herbs or medicine” from ACV teen pregnancy vignette. Artist credit: Hillary Carter-Liggett

In pilots of the ACV methodology, we developed several vignettes representing situations that are commonly understood by Tanzanian adolescents. As discussed above, the primary method of reducing risk was to present stories to young respondents on tablet computers, using audio heard privately through headphones. The stories were followed by questions about the stories conducted using a typical interviewer-led survey, but with reference to the videos that would be unlikely for nearby adults to understand (since they didn’t hear the videos). This, in turn, reduced the risk of participants being punished for responses viewed as inappropriate. Using free software and simple drawings, we created short cartoon videos with young protagonists along with recorded voice-overs in Swahili. The cartoon characters lack physical or contextual characteristics that would associate them with any particular ethnicity or socio-economic status. Figure 1 shows two of the images from the story about teen pregnancy.

As described above, respondents watched a video and then answered survey questions about the dilemma it described. Some response options used a 5-point “Smiley Scale”: respondents could point at a face emoji (very sad/angry to very happy). Other questions required responses of a word or phrase. To better understand the quantitative results, some young respondents participated in brief qualitative interviews after the conclusion of the quantitative survey data collection, and some joined sex- and age-specific focus groups. All stories were validated in collaboration with the Tanzanian field team and through cognitive interviews with Tanzanian adolescents.

In the vignette analyzed in this paper, a schoolgirl with a boyfriend finds herself pregnant. The story explains that the girl would like children at some point, but now is not the right time. The girl recognizes that it is difficult for pregnant girls and mothers to remain in school. The cartoon girl thinks about several possible outcomes for the pregnancy, including getting an abortion, marrying the boy, or asking grandparents to care for the baby. Girls heard a female voice telling the story from the point of view of the cartoon girl. Boys were shown exactly the same video images but heard a male voice narrating from the point of view of the father of the fetus. No information about the cartoon couples’ exact ages, education levels, or family backgrounds was given, though our pretesting of the story suggests most respondents interpreted the characters as young people of similar ages to themselves.

Interviewer effects on survey data are a persistent concern for researchers especially when interviewing children and adolescents [19]. In an attempt to please interviewers, respondents may answer questions in ways that are consistent with a dominant social narrative; Morris [26] calls such responses “scripts” based on her research with adolescents in Zanzibar, Tanzania. For example, Mitchell et al. [24] found that children in Kenya often referenced textbook sentiments about abortions. In our study, survey questions following the vignette asked what the cartoon characters should do. The question wording allowed the respondent to keep the conversation firmly in the hypothetical third person (about the cartoon character) instead of asking respondents to share information about their personal opinions or experiences. Although Mitchell et al. [24] found that their respondents were more understanding of peers and of themselves than of vignette characters, in our focus groups young people often used local examples or even slipped into the first person when describing what the cartoon character should do in a difficult situation. This is a local example:

*“I was studying with this girl. She got pregnant. The father of this girl came to school, and the teachers said, ‘we can’t accept this girl back because she is pregnant.’ The girl dropped out of school. But as her friends, we were not happy about the situation.”* [boy, 17, focus group, rural].

Even though we explicitly did not request information about young people’s own experiences, these came up naturally in qualitative discussions. Similar to Mitchell et al.’s [24] conclusions, we demonstrate below that Tanzanian youth express opinions that sometimes conform to but also sometimes contradict social narratives or scripts, even when discussing hypothetical vignettes.

Some of the quotes presented in this paper may make it seem as if a child were asked directly about abortion or were asked to describe personal experiences. This was not the case. During interactions between field researchers and young respondents, we aimed to minimize any discussion using the word “abortion” in order to protect the adolescent from repercussions from conversing with a stranger about a sensitive topic. As corporal punishment is common in Tanzania, ethical protection of children as a vulnerable population necessitated extra caution on behalf of the research team to mitigate the potential of a child being punished by an adult who overheard the interview [41]. Instead of speaking directly about abortions, the euphemism “take herbs and medicine to get her period back” was used in Swahili. The results section will show that most young respondents understood this euphemism. If a child voluntarily used the word “abortion” or mentioned other sensitive topics, field researchers were trained to continue the conversation only if the location of the interview was private enough that there was no risk of being overheard by adults or other children. We conducted a small follow-up study with respondents in the pilot and none reported any risk or discomfort following the interview ([21], pg S152). We attribute our success to these precautions.

### Data Collection

**Fig. 2:**
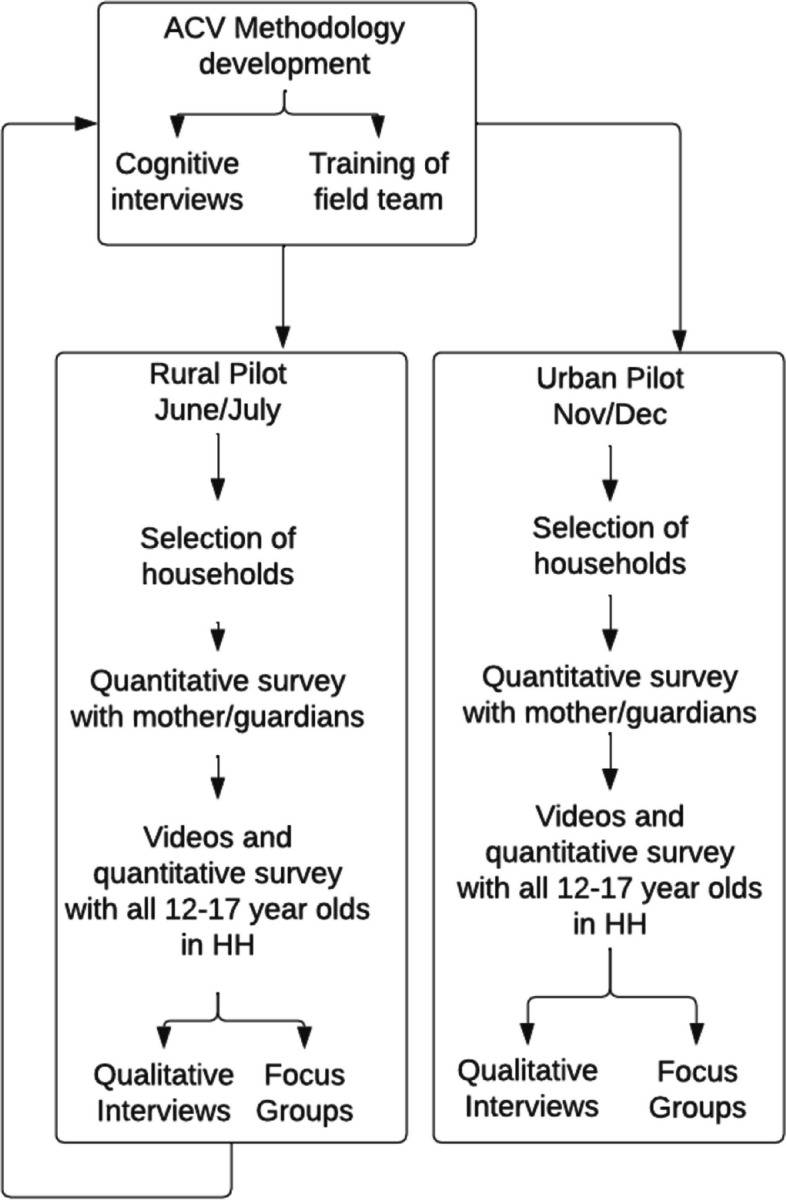
Study development and pilot studies in Tanzania 2018

The vignette methodology was piloted in two locations in northern Tanzania in 2018 using a mixed-methods approach as shown in Fig. 2. This project was approved on May 18, 2018, by the IRB of the University of Minnesota (STUDY00003131) and by the Commission for Science and Technology (COSTECH) on May 10, 2018, in Tanzania. Adult and child participants were given a small gift of sugar, school supplies or a small monetary payment based on recommendations by local collaborators. The first pilot location was a village in the Arusha District that was purposefully selected based on the diversity of ethnicities (predominantly Chagga and Iraqw), religions (Christian and Muslim) and occupations (farming, herding, and small businesses). Following the rural pilot, a second pilot was conducted in urban areas in Arusha District. We used a household-based instead of a school-based sample and did not require literacy to identify our study population. The pilots used a two-stage systematic random sampling of households in wards and neighborhoods drawn for the purpose of this study by the field team with support from local village and community leaders, as discussed in Bolgrien and Levison [6]. In each household, an adult answered a questionnaire about household demographics, and all available children ages 12–17 in the household were asked to participate in a face-to-face administered survey that included vignettes. Adults gave consent for household and child participation and children gave assent to the interviewer prior to the start of the survey. Survey teams were trained to conduct the survey in a public (visible) area but out of earshot of adults, to create privacy for the child respondents during in-person surveys and one-on-one interviews; training also included other methods to reduce perceived power disparities between adult interviewers and young interviewees [7]. Each pilot included teams of 4–6 experienced young Tanzanian interviewers; the authors and local staff conducted additional training in survey data collection and qualitative methods with adolescents.

**Table 1:** Descriptive statistics for ACV pilots in Tanzania, 2018

	Rural (June-July)	Urban (Nov-Dec)	Total
Child respondents (N)	133	194	327
Households (N)	103	145	248
Female	47%	53%	50%
Age			
12	30%	16%	22%
13	24%	24%	24%
14	16%	16%	16%
15	8%	16%	13%
16	10%	14%	13%
17	12%	13%	13%
Muslim	41%	18%	27%
Christian	59%	79%	71%
Current student	72%	94%	85%
Watched teen pregnancy video	104	187	291
Post-survey interviews	66	86	152
Number of focus groups	10	13	23
Focus group participants	46	55	101

Table 1 shows sample characteristics for young survey respondents. In total, 327 children in 248 households were surveyed. Most came from relatively large households of about six people (including themselves). In each household, each available (and assenting) 12–17 year-old was included in the survey. In both samples, especially the rural village, the sample was skewed toward younger ages. Older children were often away in boarding school or had left home to work. The urban field research was conducted during the beginning of a school holiday so more older adolescents were available. Although all survey participants had attended school at some point, more than one-quarter of the rural children were no longer enrolled in school. The vignette about teen pregnancy was only one of several possible vignette topics the children watched. Children were asked between each video if they would like to continue participating. In the rural village, children were shown up to four videos in a random order. During the rural pilot, one-third of respondents did not watch all four vignette videos, but data on the reason for discontinuing – a child’s decision, a field team member determining the child was fatigued or distracted, or an adult interrupting the interview – was not collected. Based on feedback from the field team after the rural pilot, we modified the survey design for the urban pilot to present 3 videos in a set order to reduce respondent burden. In the urban pilot, only 4% of respondents did not complete the 3 videos. A subset of 291 out of the 327 surveyed adolescents watched the story about teen pregnancy and answered its follow-up questions.

To better interpret the results from the quantitative survey data collected from households and children, we also collected concurrent qualitative data from the child respondents. After participating in the survey, a subset of 152 children assented to participate in semi-structured interviews which took place directly following the child’s survey. Children were asked about their answers to some of the survey questions about one or more of the videos. Finally, children who participated in the survey were asked if they would like to participate in focus groups. Interested and available children were organized into focus group discussions of three to 10 participants, grouped by sex and similar ages, to have conversations about the vignettes. The aim of these short interviews and focus groups was to assess the understandability of the vignettes, the degree of personal connection the children felt in regard to each story, and to explore ideas children had about possible outcomes for the story. The focus group discussions and interviews were conducted, recorded, transcribed verbatim, translated from Swahili to English by the field team, and coded using ATLAS.ti 8 (Version 8.4.24.0) [2] in a collaborative and iterative effort by both authors and a project assistant. The story about teen pregnancy was discussed in 90 of the interviews and focus groups, and the topic of abortion was discussed 87 times. Language used in this paper will attempt to be true to the respondents’ language, e.g. referring to the cartoon boy as the “father” and saying “baby” instead of “fetus.”

### Results and discussion about using a euphemism for abortion

As discussed above, we avoided using the term “abortion,” instead using the euphemism “the girl could take herbs or medicine to get her period back,” similar to other researchers’ use of euphemistic phrases like “sleep with someone” and “to make love” instead of “sexual intercourse” [33, 5]. During the preparation and training for the field work, this phrase was generally understood by respondents. We continued to validate that this phrase was understood during the qualitative interviews that followed the survey.

Based on the follow-up interviews, older respondents of both sexes understood the language around “herbs and medicine” to be referring to abortion. When abortion was discussed, 26 respondents used language that indicated their understanding that the situation implied abortion or terminating the pregnancy. For example:

*Interviewer: And then she uses herbs or medicines to get her periods back, what do you think is going to happen?*

*Respondent: abortion*

*Interviewer: what are the effects of it?*

*Respondent: The unborn baby will die.* [boy, 14, urban]

In another 13 interviews, the young respondent’s language indicated clearly that she or he understood the purpose of using herbs and medicine but did not refer to abortion directly. Instead, language such as “bringing back normal periods,” “losing the baby,” “grief,” and “negative effects” are examples of how respondents referenced the termination of a pregnancy. A 14-year-old girl indicated in the survey that the cartoon girl was somewhat happy to take herbs and medicine, and during the interview the respondent described happiness resulting from using the herbs. The interviewer asked, “what are other effects after she gets her period back?” and the girl replied, “abortion.” After this, the respondent became less talkative and responsive and changed the subject.

Seven interviewees (both genders, age 12–15) likely did not understand the nuanced language of “herbs and medicine” to imply abortion. One (boy, 13, rural) misunderstood that herbs or medicine referred to birth control or pre-natal care given at hospitals. Additionally, some younger boys and girls did not make the connection between menstruation and pregnancy. Education (formal or informal) about reproduction and reproductive health is very limited for younger children in Tanzania [27]. Whereas girls may be warned about the possibility of pregnancy when they begin menstruating, this may not happen for boys entering puberty. Several of the older boys incorrectly described female reproductive anatomy and how or when to use birth control.

#### Results and discussion on young people’s perspectives on abortion

A survey question about the teen pregnancy vignette asked respondents to identify what was most likely to happen to the cartoon kids. As shown in Table 2, among the options presented, 18% of respondents reported that the girl would abort the baby; it was the third highest-ranking option out of the five options, behind getting married and taking the baby to the girl’s family. In an open-ended question (not shown) that asked the respondent to imagine the most likely outcome to the story if it happened “around here,” 11% chose abortion.

**Table 2:** Results to quantitative survey question: What should the cartoon kid do? (Options presented in video)

	Male		Female		Total	
	n	(%)	n	(%)	n	(%)
“Take herbs, get period back” (abort)	26	17.2	26	18.7	52	17.9
Get married	50	33.1	38	27.3	88	30.3
Boy’s parents care for baby	33	21.9	6	4.3	39	13.4
Boy denies baby	22	14.6	5	3.6	27	9.3
Girl’s parents care for baby	20	13.2	63	45.3	83	28.6
Total	151	100	138^a^	100	289^a^	100

Pearson chi^2^ = 52.83 Pr = 0.00

^a^One girl did not provide an answer to this question

The relative popularity of the options about getting married or having parents of the girl or boy help to care for the baby is consistent with the qualitative findings from the interviews and focus groups. Respondents often described the cartoon girl and boy as considering possible outcomes in order of desirability: If it was unlikely the cartoon couple could marry or care for the baby themselves, they would next approach one or both sets of parents; if that was unsuccessful or undesirable, then an abortion was considered. For example,

*She will feel happy because it’s something she did not really like and did not expect it. So, if he* [presumably the cartoon boyfriend] *goes to his parent, first if he goes to the parents of the girl. I mean they can reject* [the pregnancy or baby] *and they can hate him. So she was, I mean … that’s why I said she would feel happy. Because, I mean, she probably can’t afford it and she might need to reduce her responsibilities. Because if she used those herbs to abort the pregnancy, you will find that she can continue with her normal things.* [boy, 14, urban, 5 on Smiley Scale]

When we asked young respondents how they felt about abortion using the Smiley Scale, 56% of them thought abortion was a negative outcome (sad/angry or very sad/angry) for the cartoon couple, as shown in Table 3. An additional 18% reported a neutral feeling (3 on the Smiley Scale). 25% reported that abortion would be a positive outcome (happy or very happy). Girls were less likely to be very happy than boys and more likely to be very sad/angry, but a chi-square test for the Smiley Scale cross tabulation by gender was significant only at the 11% level. T-tests for differences in the specific Smiley Scale responses by gender were significant only for “very happy” (*P* = 0.012, not shown). The results from the qualitative analysis of interviews and focus groups helped us understand these quantitative results.

**Table 3:** Responses to survey question: How does the cartoon girl/boy feel about the option [“take herbs or medicine to get her period back”]?

	Male		Female		Total	
	n	(%)	n	(%)	n	(%)
Very sad/angry (1)	43	28.5	52	37.4	95	32.8
Sad/angry (2)	38	25.2	32	23.0	70	24.1
Neither (3)	26	17.2	27	19.4	53	18.3
Happy (4)	22	14.6	20	14.4	42	14.5
Very happy (5)	22	14.6	8	5.8	30	10.3
Total	151	100	139	100	290	100

Pearson chi^2^ = 7.53 *P* = 0.11

Of the respondents who indicated that abortion was a positive outcome, the primary reason mentioned was that an abortion would allow the cartoon girl to return to her normal life and possibly stay in school or go back to school.

*She may feel happy because, if you have not got your periods for a while, it could be a problem. So, it is better to look for herbs/medicines that will help. She has to deal with that in order to get her periods back.* [girl, 17, urban, 5 on Smiley Scale]

*She has very big dreams in her life. She has dreams that will take her five years to reach: ‘I want to be a certain type of person later.’ The boy came and shortened her dream. She will make sure… she aborts the pregnancy so that she achieves her dreams…* [girl, focus group ages 14-17-year-old, rural]

These positive reactions are consistent with strong social norms in Tanzania for children to complete their education before starting a family [44]. Based on the qualitative evidence, both girls and boys felt that the cartoon girl should be in school, and abortion was the mechanism that would allow that.

Many of the reasons given for a negative reaction to abortion were consistent with moral and ethical qualms associated with it. In our study, children said that ending the pregnancy will make the girl sad because “the baby will die” (multiple respondents). Other reasons for negative responses towards abortion reflected the dangers of illegal abortion services, particularly in rural areas where undergoing abortions may be especially risky for the mother. Mitchell et al. [24] noted that such dangers, while real, are greatly exaggerated in Kenyan schools and educational materials; this may also be true in Tanzania.

*She feels sad because she might die in the process of abortion, that is why she will feel very sad.* [girl, 14, urban, 2 on Smiley Scale]

Given the strong social norms of remaining in school and cultural stigmatization of teen pregnancy and abortion in Tanzania, the results of strong negative and strong positive opinions found in the quantitative smiley-scale and accompanying qualitative validation are not surprising. But why would a respondent feel neutrally about abortion? In follow-up interviews, some respondents described the complexity of a situation that could involve abortion. One older boy said:

*When I said abortion, I assumed the boy denies the pregnancy and leaves the girl to decide on her own. The girl can abort the pregnancy so as to look like other girls…She can go to school and feel young as other girls. She will decide to abort the pregnancy so she can match with other girls of her age and also* [have the abortion] *when the pregnancy is not noticeable to other people. Most girls end up doing this when the boys deny the pregnancies.* [boy, 17, urban, 3 on Smiley Scale]

The cartoon boy denying that the baby was his and abandoning his girlfriend seemed likely to this respondent, who chose abortion to spare the cartoon girl from stigma and other troubles. Because he saw both negatives (denying paternity, abandonment) and positives (being able to hide the pregnancy), he chose the middle Smiley emoji.

#### Results and discussion on healthcare access and gendered decision-making

Survey results show that there is variation in how children and adolescents are thinking about cultural norms, social stigma, and the choices about hypothetical, yet possible, decisions that they or their peers may face in their own communities. The findings from the ACV methodology show that young people understand and interpret teenage pregnancy as a complex situation with multiple overlapping expectations, including staying in school and abstaining from sex while in school. Two additional themes appeared during the qualitative conversations about abortion: unaffordable health care and gendered decision making. Both of these topics are central to broader conversations about sexual and reproductive health access and female empowerment in Tanzania.

Respondents were acutely aware of the life-threatening nature of illegal abortions, particularly using local medicine or witch doctors as opposed to costly private clinics. One girl (age 17, urban) spoke of hearing about a girl going to a witch doctor for an abortion, but it was unsuccessful and resulted in an infection. The expense of an abortion was often directly connected to discussions about who would decide whether the fetus would be carried to term.

In the quantitative survey respondents indicated whom they thought would be the decision maker in the vignette about pregnancy. Table 4 shows that almost half of surveyed participants (48%; including 50% of girls and 46% of boys) reported the cartoon boy would make the decision and only 17% thought it would be the cartoon girl; only 3% thought the boy and girl would make the decision together. Other young respondents thought that adults such as parents or leaders (31%) or a combination of adults and adolescents (1%) would make the decision.

**Table 4:** Results to quantitative survey question: Who will decide what happens?

	Male		Female		Total	
	n	(%)	n	(%)	n	(%)
(If cartoon boy): Boy^a^	58	38.4	7	5.1	65	22.5
(If cartoon boy): Girl^a^	16	10.6	5	3.6	21	7.3
(If cartoon girl): Girl^a^	1	0.7	27	19.6	28	9.7
(If cartoon girl): Boy^a^	11	7.3	63	45.7	74	25.6
Boy’s parents	19	12.6	4	2.9	23	8
Girl’s parents	24	15.9	15	10.9	39	13.5
Kid & parents	1	0.7	2	1.4	3	1
Leaders	3	2	1	0.7	4	1.4
Boy’s & girl’s parents	14	9.3	9	6.5	23	8
Boy and girl together	4	2.6	5	3.6	9	3.1
Total	151	100	138^b^	100	289^b^	100

Pearson chi^2^ = 17.85 Pr = 0.47

^a^Respondents were supposed to watch the video that showed a cartoon character that corresponded to their own gender, but this was not always the case as shown above where 12 male respondents answered questions about the cartoon girl, and 12 female respondents answered questions about the cartoon boy

^b^One girl did not provide an answer to this question

Many conversations in the qualitative interviews and focus groups talked about gender roles and decision-making when considering the cost of abortion and safe access to the healthcare system. The father of the baby was typically expected to determine whether to abort, either via positive support (the contribution of funds to pay for the abortion) or negative coercion (abandoning the cartoon girl and forcing her to make a decision between single motherhood or abortion).

For example, here is an excerpt from a focus group discussion among boys ages 13 and 14 [rural]:

Boy 1: *He* [another boy in focus group] *says that the girl is the one to decide about aborting the pregnancy because the pregnancy is within her body. She is the one with the decision of abortion because that boy, even if he can manage to tell her to abort the pregnancy, she is the one with the decision. She can make the decision to go to search for drugs, get them, and abort that pregnancy.*

Boy 2: *I think that will not be possible, because where will she get the money? And you might find that her parents are against her. She cannot do it. They* [the girl and boy] *will have to sit together and negotiate about the pregnancy…They have to say whether they are getting married, should get married. If they have to abort, then abort…But one of them cannot get the ideas* [alone] *because they will go into the wrong direction.*

Other respondents described the father of the unborn baby – the cartoon boy – as being the head of the family. They felt it was therefore up to the cartoon boy to find money for the girl and to help the girl decide what to do about the pregnancy. Both girls and boys took this patriarchal position. For example:

*The boy has the power to do anything and also he is the head of the family. Probably, this girl wouldn’t be able to raise the baby or provide the basic needs for the baby.* [girl, 12, rural]

*The cartoon boy is the one with decisions to abort the pregnancy because he is the one who knows how he will take care for the child or how he will raise it.* [boy, focus group ages13-14, rural]

Conversely, many girls (and some boys) were more likely to assert that the cartoon *girl* had the final decision about what to do about the pregnancy since the girl was the one carrying the baby and the one who faced the most risks if the pregnancy or abortion went wrong. Conversations in some female focus groups described situations where the cartoon girl would be able to find the money from her parents or by working. Some girls were also acutely aware that the final decision would often be left to the cartoon girl if the boy abandoned her or decided to deny that the baby was his, as in this case:

*She will have thought ‘Maybe this boy will reject me? Will I fail to raise my child?’ Maybe she will also go to people who will advise her to abort the pregnancy so that she can go back to how she used to be. [girl, 17, urban]*

As shown in Table 4, while half of the girls responded that the cartoon boy would make the decision about the outcome of the story, 23% of the female respondents reported that the cartoon girl would be the final decision maker. This was the second most popular result for female respondents whereas the cartoon girl’s parents was the second most popular option (16%) for male respondents. Excluding the cartoon girl from the decision regarding her own pregnancy reflects the strong patriarchal norms in Tanzanian society (Leshabari et al. [20]).

## Conclusion

Both our study and Mitchell et al. [24] suggest that East African children and adolescents have complex understandings about pregnancy and abortion, with discourse that may be heavily influenced by religious or other teachings but with pragmatic, less-judgmental responses to actual situations. These results show that there is variation in how children and adolescents are thinking about cultural norms, social stigma, and the choices about hypothetical, yet possible, decisions that they or their peers may face in their own communities.

Before exploring the results on views on abortion, stigmatization, access, and decision making, we first needed to confirm that the majority of children in the study understood the phrase “take herbs and medicine to get her period back” to be a euphemism for having an abortion. A limitation in our study is that we did not test directly using the word “abortion” because that would have exposed young respondents to increased risk of harm. This methodological innovation was successful as young respondents’ use of language indicated an awareness about social norms when discussing a sensitive topic with researchers (adult strangers). Participants likely considered their comfort level about interacting with the field researcher, as well as the likelihood of others overhearing the conversation. While our team took many precautions to avoid increasing risk to respondents, including monitoring the environment during the interview for anyone who might be listening, respondents were also actively making decisions about what language they used and their levels of openness in their responses. We argue that the benefits of being able to ask vulnerable populations questions about a sensitive subject are worth potential misunderstandings by a small minority of participants.

The findings from the ACV methodology show that young people understand and interpret teenage pregnancy as a complex situation with multiple overlapping expectations, including staying in school and abstaining from sex while in school. Our findings on the mixed positive and negative perspectives of abortion in the quantitative survey would have been difficult to interpret without the qualitative interviews. The video vignette methodology in this study successfully engaged with young people about a complex and sensitive topic in ways that showed that adolescents could formulated complex and thoughtful opinions on such topics. Young people’s quantitative answers to survey questions and qualitative comments reflected the lack of safe and affordable options and services for youths. Even though abortion was clearly perceived as physical risky and socially stigmatized, both girls and boys promoted it as a solution that would leave the cartoon girl in school, where they felt she belonged. An early, secret abortion could protect the girl from being expelled from school. However, respondents indicated that the cartoon boy must participate in this scheme because girls on their own were not expected to have the resources to seek a lower-risk abortion.

Even at a young age, girls and boys are balancing the high risk of unsafe abortions against the benefits of keeping the pregnancy secret and avoiding social stigmatization for either pregnancy or early school-leaving. In our sample, respondents of all ages and both genders discussed abortion with maturity and understanding. Boys’ perspectives are infrequently included in studies about abortion or reproductive health care services, but the findings from this mixed methods study show that boys (and girls) are considering the nuanced role of boys and men in supporting or funding an illegal abortion. Framing the decision of an abortion as involving not only the pregnant girl but also other influential people such as the boyfriend or the girl’s parents can inform sexual and reproductive health policies to better support young people. However, the sample sizes in these pilot studies are too small to identify patterns correlated with demographic characteristics.

The secrecy and stigmatization of teen sexual relationships and illegality of abortion have made it very hard for researchers (let alone policymakers) to understand how adolescents are weighing trade-offs and navigating the competing pressures from peers, relationships, and familial expectations about education. Using the Animating Children’s View methodology, the sensitive topic of abortion was discussed with adolescents and children as young as 12 in a way that allowed them to share their perspectives without divulging personal information about sexual behaviors. Video cartoons engaged their attention leading to serious questions and sometimes conversations about hypothetical cartoon characters. In a context where pregnancy and motherhood mean the end of schooling for girls, respondents grappled with difficult decisions the cartoon couple faced regarding pregnancy and potential abortion. In future research, it will be imperative to explore adolescents’ perspectives regarding abortion for schoolgirls as it relates to increased access to education for pregnant girls. The ACV methodology can be used to engage adolescent girls and boys about pregnancy, abortion, contraceptive access, and cultural norms and stigma so their voices are included in such policy decisions.

## Data Availability

Participants of this study did not consent for their data to be shared publicly.

## Abbreviation

ACV: Animating Children’s Views
